# Perceived Control and Cognitive Aging: The Mediating Role of Mediterranean Diet Adherence

**DOI:** 10.1093/geroni/igaf122.2908

**Published:** 2025-12-31

**Authors:** Kelly Parker, Jeremy Hamm

**Affiliations:** North Dakota State University, Fargo, North Dakota, United States; North Dakota State University, Fargo, North Dakota, United States

## Abstract

Consistent evidence has shown that perceived control (PC) buffers against age-related cognitive declines over periods of up to 20 years. However, little is known about how core health behaviors (e.g., diet) and demographic factors (e.g., socioeconomic status (SES)) may mediate and moderate this relationship. Using national data from the Midlife in the United States (MIDUS) study (n = 831, age range = 34-83), this study examined the role of a Western Mediterranean Diet (wMD) as a mediator between, and SES as a moderator of, PC and cognitive functioning. Participants provided complete data for age, sex, education, income, race, diet, and health locus of control (PC) at Wave 2, as well as 9-year longitudinal data on episodic memory (EM) at Waves 2 and 3. Hayes’ PROCESS Macro was utilized to identify how the relationship between PC and longitudinal EM may be mediated by wMD and moderated by central indicators of SES (income, education). Step 1 mediation models showed that wMD mediated the association between PC and 9-year episodic memory (b = .0084, CIs = .0002-.0211). Step 2 moderated mediation models showed that this wMD mediated pathway was pronounced for only those with less education (b = .0216, CIs = .0045-.0469). Results advance the literature by providing new evidence for (a) the mechanistic health behavior processes (wMD) that account for the relationship between PC and episodic memory and (b) the sociodemographic populations for whom this mediated pathway is most pronounced (those with lower SES).

